# Classification and Identification of Frequency-Hopping Signals Based on Jacobi Salient Map for Adversarial Sample Attack Approach

**DOI:** 10.3390/s24217070

**Published:** 2024-11-02

**Authors:** Yanhan Zhu, Yong Li, Tianyi Wei

**Affiliations:** 1 School of Electronics and Information Engineering, Nanjing University of Information Science and Technology, Nanjing 210044, China; 202312490292@nuist.edu.cn; 2 Sixty-Third Research Institute, National University of Defense Technology, Nanjing 210007, China; liyong63s@nudt.edu.cn

**Keywords:** deep neural network, adversarial sample, classification and identification of frequency-hopping signals, Jacobi saliency map, no-target attack

## Abstract

Frequency-hopping (FH) communication adversarial research is a key area in modern electronic countermeasures. To address the challenge posed by interfering parties that use deep neural networks (DNNs) to classify and identify multiple intercepted FH signals—enabling targeted interference and degrading communication performance—this paper presents a batch feature point targetless adversarial sample generation method based on the Jacobi saliency map (BPNT-JSMA). This method builds on the traditional JSMA to generate feature saliency maps, selects the top 8% of salient feature points in batches for perturbation, and increases the perturbation limit to restrict the extreme values of single-point perturbations. Experimental results in a white-box environment show that, compared with the traditional JSMA method, BPNT-JSMA not only maintains a high attack success rate but also enhances attack efficiency and improves the stealthiness of the adversarial samples.

## 1. Introduction

In the increasingly complex electromagnetic environment, frequency-hopping communication systems, known for their robust anti-interference, anti-fading, and multiple access networking capabilities, play a crucial role not only in civilian communication but also in the defense and military sectors, where they have achieved remarkable success [1]. Consequently, the effective identification and classification of frequency-hopping communication signals has become a key research focus within the field of signal processing. With the rapid advancement of electronic countermeasure technologies, the demands for greater reliability and stability in frequency-hopping communication have intensified, and the inherent limitations of traditional systems are becoming increasingly apparent [2]. In communication countermeasures, reconnaissance equipment can frequently detect and capture multiple frequency-hopping signals originating from various independent radio stations. To gain a tactical advantage on the battlefield, it is essential to classify and identify these mixed signals within a limited reconnaissance time-frame.

Traditional methods for classifying and recognizing frequency-hopping (FH) signals typically rely on manually extracted or self-learned features from the time and frequency domains. In [3], a method is proposed for classifying and identifying single FH stations by extracting the transient characteristics of FH signals. This approach achieves a higher classification and recognition rate for a single FH station compared to classical template matching methods, with an average recognition accuracy of 92.6%. In [4], a method is introduced that combines the K-means clustering algorithm with hop periods to sort non-blind signals across different networks. Building on this, additional parameters such as the direction of arrival, power, and hop time are employed to further sort stations within a network. In [5], the author extracts fingerprint features representing emitter signals and uses a decision tree model within an integrated learning framework to effectively classify and recognize these signals.

In recent years, the successful application of deep learning in fields such as image [6] and voice recognition [7] has prompted researchers to explore its use in wireless communication, particularly for FH signal classification and recognition. For instance, in [8], the author innovatively applies principles from target detection in image recognition to address the problem of radio signal recognition, significantly enhancing both recognition performance and the intelligence level of signal processing in complex electromagnetic environments. In [9], a three-layer sparse autoencoder is used to extract key features from modulated signals, followed by a backpropagation (BP) neural network to identify communication signal modulation modes. The method achieves a classification accuracy of 98% across a signal-to-noise ratio (SNR) range of 0–20 dB. Lastly, in [10], the author conducts time-frequency analysis of FH signals, extracts contour information to construct a contour map, and uses a convolutional neural network (CNN) to classify and recognize FH signals.

Deep learning technology has demonstrated outstanding performance in the classification and recognition of frequency-hopping signals, significantly enhancing both accuracy and efficiency [10]. In electronic countermeasure environments, adversaries can leverage deep neural networks to classify and identify intercepted frequency-hopping signals, enabling them to implement targeted jamming strategies. Such strategies pose a serious threat to the information security of communicators. Since 2014, Szegedy [11] and other researchers have highlighted the existence of adversarial samples, which have been shown to induce deep learning models to make incorrect predictions. Given that frequency-hopping signals often contain higher levels of noise and uncertainty, they are particularly susceptible to the influence of adversarial samples when deep learning is applied directly for classification and recognition. As a result, research into the generation of adversarial samples capable of deceiving enemy classifiers holds significant practical value.

Currently, adversarial sample generation methods [12,13,14,15,16,17,18] in the field of electromagnetic signals predominantly adopt global attack strategies. These approaches often fail to adequately account for the local characteristics of the signals when implementing adversarial perturbations, thereby limiting the effectiveness of the attacks. To address these limitations and to generate higher-quality adversarial samples for communication signal attacks, this paper introduces a method for generating adversarial samples for frequency-hopping signal classification, inspired by the Jacobian-based saliency map attack (NT-JSMA) [12], originally developed for image recognition. The proposed method significantly enhances the speed of adversarial sample generation by selectively targeting key feature points on the frequency-hopping signal feature map for perturbation. Moreover, the method restricts the selection of feature points to ensure that the generated adversarial samples remain more concealed. The main contributions of this paper can be summarized as follows:

1. This experiment demonstrates the limitations of both the conventional gradient attack method and the JSMA method in attacking classification and recognition models for frequency-hopping signals. In response to the non-stationary characteristics of frequency-hopping signals, a new method—BPNT-JSMA—has been proposed to generate adversarial samples specifically tailored for the classification and recognition of such signals.
2. The BPNT-JSMA method generates a feature saliency map of the frequency-hopping signal by computing the Jacobian matrix. It then selects, in batches, the feature points with the highest saliency to introduce perturbations, thereby producing adversarial samples. This approach significantly accelerates the generation of adversarial samples compared to the conventional targetless JSMA method.
3. The BPNT-JSMA method introduces a clipping function, which is not available in the NT-JSMA method, and adds $L_{\infty }$ restriction to ensure that the perturbation values added to the samples do not exceed a certain range, thus enhancing the stealthiness of the generated adversarial samples.

Section 2 of this paper provides a systematic overview of the fundamental concepts of adversarial samples and the underlying principles of adversarial sample generation methods. Section 3 presents a detailed description of the BPNT-JSMA adversarial sample generation method, which is based on salient graphs for batch feature points. This section also offers an in-depth theoretical analysis of the proposed method. In Section 4, experimental validation is carried out. The experimental setup is first described, followed by the introduction of evaluation metrics for the algorithm. Subsequently, a series of experiments are conducted, and the results are thoroughly analyzed. Section 5 concludes the paper by summarizing the findings and discussing potential directions for future research.

## 2. Related Literature Review

### 2.1. Basic Concepts of Adversarial Samples

Adversarial samples are crafted by introducing small, often imperceptible perturbations to the original data set. While these modifications may go unnoticed by a human observer, they can significantly alter the model’s output, leading to incorrect predictions. Such samples are highly stealthy and are specifically designed to exploit vulnerabilities in the model, causing it to confidently misclassify the input. The process of generating adversarial samples can be described as follows:

(1) $$x^{adv}=x+\arg\min||\delta |{|}_{p}$$

(2) $$F(x^{adv})\neq F(x)$$

where $x^{adv}$ is the adversarial samples, δ is the adversarial perturbation, p is the paradigm constraint of the adversarial perturbation δ, F is the deep learning model, and x is the original sample.

### 2.2. Adversarial Sample Generation Methods

#### 2.2.1. FGSM

Fast Gradient Sign Method (FGSM) [13] and its variants [14,15,16,17] are currently the most efficient and widely used methods for generating adversarial samples, which maximizes the loss function by adding a perturbation in the opposite direction of the gradient to generate adversarial samples that induce the model to produce erroneous outputs. With its implementation, the disadvantage is that its attack mode is global attack, and the generated adversarial samples are insufficient in terms of concealment.

In 2015, Goodfellow et al. [13] proposed the most classical gradient attack algorithm, the Fast Gradient Sign Method (FGSM), based on the assumption of linearity of neural networks in high-dimensional space. The adversarial sample generation algorithm of FGSM is shown in Equation (3).

(3) $$x^{adv}=x+\varepsilon \cdot sign(\nabla_{x}L(x,y;\theta ))$$

where x is the original sample, $x^{adv}$ is the generated adversarial sample, y denotes the correct label of the original sample x, θ is the parameter of the deep learning model, ε denotes the size of the added perturbation, L(⋅) is the loss function, $\nabla_{x}$ is the derivation of the loss function (the gradient information of the original sample), and sign(⋅) denotes the sign function. The most important feature of the FGSM is its high efficiency, which is often used in scenarios that require a large number of generated adversarial samples such as adversarial training. The most notable feature of the FGSM is its efficient operational speed, making it widely used in scenarios that require a large number of generated adversarial samples, such as adversarial training. However, a notable drawback is that the performance of the generated adversarial samples is generally inferior.

#### 2.2.2. I-FGSM

In order to overcome the limitations of FGSM, Kurakin et al. [14] proposed the Basic Iterative Method (BIM) in a follow-up study. BIM is also known as Iterative Fast Gradient Sign Method (I-FGSM), and its core strategy is to change the single-step addition of perturbations in FGSM to a small number of times, i.e., to add small perturbations along the direction of the gradient increase in each iteration, to observe the change in the model’s decision-making, and to return to the adversarial samples once there is an error; the equations are shown in Equations (4) and (5).

(4) $$x_{0}^{adv}=x$$

(5) $$x_{i+1}^{adv}=Clip_{x,\varepsilon }\{x_{i}^{adv}+\alpha \cdot sign(\nabla_{x}L(x_{i}^{adv},y;\theta ))\}$$

where clip{·} is the cropping function, which ensures that the pixel values of the adversarial samples are in the appropriate range; $x_{i}^{adv}$ is the adversarial samples generated in the i iteration, and α is the single-step iteration size. Compared with FGSM, I-FGSM can construct more accurate perturbations and improve the quality of the adversarial samples, but the computation amount increases.

#### 2.2.3. MI-FGFSM

Based on the FGSM framework, Dong et al. [15] cleverly integrated the concept of momentum in physics, and then proposed the momentum iterative fast gradient sign method (MI-FGSM). While retaining some gradients in the previous step, they introduced a small amount of gradients generated in the current step to stabilize the update direction and avoid falling into local extreme points. The improvement of this method is to accumulate the velocity vector in the gradient direction by using momentum. Equations are shown in (6) and (7):

(6) $$g_{i+1}=\mu \cdot g_{i}+\frac{\nabla x_{i}^{adv}J(x_{i}^{adv},y)}{||\nabla_{x_{i}^{adv}}J(x_{i}^{adv},y)|{|}_{1}}$$

(7) $$x_{i+1}^{adv}=x_{i}^{adv}+\alpha \cdot \mathrm{sign}(g_{i+1})$$

First, input ${x^{adv}}_{i}$ to the classifier f to obtain the gradient $\nabla_{x}J({x^{adv}}_{i},y)$. Use Formula (6) to accumulate the velocity vector in the gradient direction to update $g_{i+1}$, and then use Formula (7) to update ${x^{adv}}_{i+1}$, and finally generate disturbance. Compared to FGSM and I-FGSM, MI-FGSM generates adversarial samples that are more aggressive and migratable.

#### 2.2.4. PGD

Mardy et al. [18] proposed Projected Gradient Descent (PGD), which, unlike the cropping operation of I-FGSM, limits the size of the perturbation by projecting the results of each iteration onto the domain of pure inputs. Compared with the one-step countermeasure of FGSM, PGD also adopts a ‘small steps, many steps’ strategy to attack by accumulating small perturbations through multiple iterations. PGD uses a uniform random noise initialization to project the gradient instead of limiting the cropping. The attack process is shown in Formula (8).

(8) $$x_{t+1}^{adv}=proj_{x,\varepsilon }(x_{t}^{adv}+\alpha \cdot sign(\nabla_{x}J(x_{t}^{adv},y,\theta )))$$

where $proj_{x,\varepsilon }(\cdot )$ is the projection operation.

#### 2.2.5. JSMA

In order to minimize the number of alterations to the original data points, researchers have introduced the Jacobian-based saliency map attack (JSMA) method [19] and its variants [12]. The methods for salient target detection only require inputting the original image and iteratively adding perturbations to the salient feature regions to generate adversarial samples without exploiting the gradient information of the model [20], which belongs to the $L_{0}$ (percentage of the number of perturbed data points) limiting attack, i.e., altering as few data points as possible.

JSMA serves as an effective adversarial attack method whose main objective is to mislead or deceive classification models such as deep neural networks for image recognition. Compared to other widely used white-box attack methods, JSMA is characterized by the innovative introduction of saliency graphs. Originally conceived to visualize the prediction process of classification models [21], in reference [19], Papernot et al. first proposed a saliency map for adversarial attacks, from which the information needed by a neural network classifier to classify a target on the sample data can be obtained. If an attacker wants to misclassify the original datum x so that it classifies t≠label(x), then it needs to strengthen all the features of t≠label(x) while shrinking the features of t=label(x) until it reaches the condition that it can fulfill the attack goal. The salient graph is shown in Formula (9).

(9) $$S(x_{\left(i\right)},t)=\left\{\begin{array}{l} 0,\frac{\partial F_{t}(X)}{\partial X_{i}}\langle \mathrm{or}\sum_{j\neq t}\frac{\partial F_{j}(X)}{\partial X_{i}}\rangle \\ (\frac{\partial F_{t}(X)}{\partial X_{i}})\cdot \left|\sum_{j\neq t}\frac{\partial F_{j}(X)}{\partial X_{i}}\right|,\;\mathrm{else} \end{array}\right.$$

where i is the input feature. First, the negative derivative classification of the specified target and the positive derivative classification of other targets are excluded. $\frac{\partial F_{t}(X)}{\partial X_{i}}$ is positive at this time, so that when the feature pixel $x_{i}$ increases, the probability value of $F_{t}(X)$ also increases, and $\sum_{j\neq t}\frac{\partial F_{j}(X)}{\partial X_{i}}$ needs to be negative or unchanged, and then $S(x_{\left(t\right)},c)$ is obtained by using all the remaining derivative components. Through $S(x_{\left(t\right)},c)$, all the input features can be easily compared, and the target can be attacked by continuously increasing these features.

JSMA enables the generation of adversarial samples that are classified as a specified target category by altering the pixel characteristics of the attack target to achieve an extreme value. In reference [12], the author introduced NT-JSMA, a variant of JSMA that does not require a predefined target class. NT-JSMA eliminates the dependency between the target and the category, allowing for untargeted attacks. However, due to the complexity of calculating the Jacobian matrix and the significant resource consumption involved, this method results in slow generation of adversarial samples. Currently, research on the JSMA algorithm primarily focuses on significantly accelerating the generation of adversarial samples while minimizing the number of pixel alterations in the attack target to achieve the desired attack effect.

## 3. BPNT-JSMA Method for Batch Feature Point Non-Target Attack Based on Jacobi Saliency Map

### 3.1. Basic Idea

**Figure 1:**
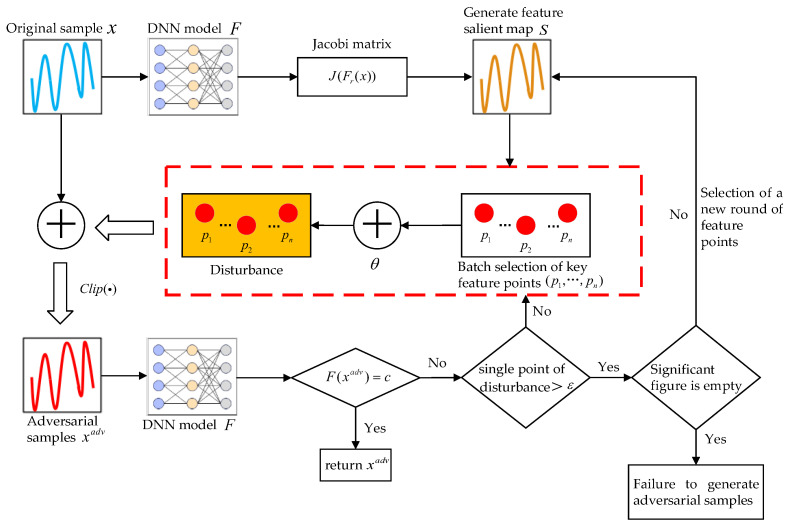
BPNT-JSMA attack flow chart.

To address the challenges associated with the JSMA method in generating adversarial samples for frequency-hopping signals, this paper proposes the BPNT-JSMA method. First, obtain the parameter information of the trained DNN model F, input the frequency-hopping signal sample x to the model F, and calculate the Jacobian matrix J for all categories. Subsequently, generate the feature saliency map according to the c of other categories, combined with the Jacobian matrix. Finally, select a subset of feature points exhibiting the strongest saliency in batches, according to the feature saliency map of the other categories c, to introduce perturbations and obtain $x^{adv}$. If the perturbations result in the model F recognizing the sample $x^{adv}$ as belonging to category c, the generation of adversarial samples is considered successful, and the adversarial samples are returned. If not, apply iterative perturbations to the feature points and evaluate whether the perturbation at each point reaches the predefined upper limit ε. If the upper limit is reached, select the next most significant feature point and repeat the process until an adversarial sample is successfully generated. If the available feature points in the saliency map allow the model F to recognize them as adversarial samples $x^{adv}$ of other categories before they are exhausted, the generation of adversarial samples will be successful. Conversely, if the feature points are depleted without successful generation, the attempt will be deemed a failure. The specific attack process is shown in Figure 1.

### 3.2. Description of the Attack Method

#### 3.2.1. Jacobian Matrix Calculation

In deep learning, the Jacobian matrix is commonly utilized to compute gradients within the backpropagation algorithm and to update model parameters. By calculating the Jacobian matrix of the output variable with respect to the input variable, we can accurately assess the impact of the input variable on the output variable. A larger value of the Jacobian matrix indicates a greater influence of the input on the output. Let the model F be an n classification model, and the dimension of the input variable x is a×b; then, obtain the score of the model F with respect to the input x for each category as the starting point, and calculate the Jacobian matrix of x for n categories according to Formula (10).

(10) $$J(F_{r}(x))=\frac{\partial F_{r}(x)}{\partial x}={\left[\frac{\partial F_{r}(x)}{\partial x_{i}}\right]}_{i\in [1,a\times b]}$$

where the result of $J(F_{r}(x))$ is the contribution of the location data point to $F_{r}(x)$,$F_{r}(x)$ represents the score of input x on the category r, $x_{i}$ represents the i feature point in x, and x has a×b feature points in total.

A deep neural network is a model composed of multiple layers of neural networks. Each layer aims to extract useful features or information from the input data and subsequently passes this information to the next layer for higher-level abstraction or processing. Most of these layers, particularly the intermediate layers, are referred to as “hidden layers”. Their inputs and outputs are not directly visible or interpretable; instead, they are automatically adjusted and optimized through the network’s learning process. Consequently, it is essential to utilize the chain rule of functions to calculate the Jacobian matrix, as demonstrated in Formulas (11) and (12).

(11) $$\frac{\partial F_{r}(x)}{\partial x}=\frac{\partial F_{r}(x)}{\partial H(x)}\frac{\partial H(x)}{\partial x}$$

(12) $$\frac{\partial H_{k}(x)}{\partial x_{i}}=[\frac{\partial f_{k}(W_{k}H_{k-1}+b_{k})}{\partial x_{i}}]$$

where $H_{k}(x)$ represents the k hidden layer, w represents the weight of the k−1 layer output as the k layer input, $f_{k}$ represents the activation function, and $b_{k}$ represents the offset.

#### 3.2.2. Saliency Map Generation

According to the aforementioned formula, the Jacobian matrix J for all categories can be derived. Subsequently, it is necessary to compute and generate the saliency map S for all categories except the true category. The generation process of the saliency map is divided into two directions: perturbation addition and perturbation reduction. In the direction of perturbation addition, larger values of significant feature points correspond to higher classification scores for the target class, while the scores for the relative non-target class decrease accordingly. In this scenario, the influence of the feature points on the classification result of the target class is positive. Conversely, in the direction of perturbation reduction, larger values of significant feature points result in lower classification scores for the target class and higher scores for the relative non-target class. Here, the influence of the feature points on the classification result of the target class is negative. Considering the effects of these two directions, Formula (13) can be derived. The key feature points that enable x to be classified as other class c can be obtained through Formula (13), which generates the feature salient graph S(x,c) of x for c.

(13) $$S(x,c)[i]=\left\{\begin{array}{cc} J_{ic}(x)|\sum_{r\neq c}J_{ir}(x)|, & J_{ic}(x)>0\wedge \sum_{r\neq c}J_{ir}(x)<0\\ 0, & J_{ic}(x)=0\wedge \sum_{r\neq c}J_{ir}(x)=0\\ J_{ic}(x)\sum_{r\neq c}J_{ir}(x), & J_{ic}(x)<0\wedge \sum_{r\neq c}J_{ir}(x)>0 \end{array}\right.$$

where i represents the i-th feature point of input x, and $J_{ic}(x)$ represents the value of the Jacobian matrix of x’s score in other c categories to the i feature point. $J_{ic}(x)>0$ means that the contribution of feature point i to the classification of x as c is positive, that is, increasing the value of i will increase the score of x in the category c; $\sum_{r\neq c}J_{ir}(x)<0$, which means that the total contribution of i to the correct category is negative; that is, increasing the value of i will reduce the score of the correct category. $J_{ic}(x)>0$ and $\sum_{r\neq c}J_{ir}(x)<0$ indicate that the contribution of the modified point to the classification of c is positive; $J_{ic}(x)<0$ and $\sum_{r\neq c}J_{ir}(x)>0$ show that the increase in the i value would lead to a decrease in the c score.

In order to increase the probability of classifying x as c, for the characteristic points of $J_{ic}(x)>0$ and $\sum_{r\neq c}J_{ir}(x)<0$, the direction of adding disturbance value is positive. For the characteristic points with $J_{ic}(x)<0$ and $\sum_{r\neq c}J_{ir}(x)>0$, the direction of adding disturbance value is negative. By performing the above calculation for each feature point in x, the feature saliency map S(x,c) can be obtained.

#### 3.2.3. Adversarial Sample Generation

After obtaining the saliency map for category c, the traditional JSMA method iteratively selects the pair of points with the highest saliency $\left(p_{1},p_{2}\right)$ to introduce perturbations and generate adversarial samples. To optimize this process and enhance the generation rate of adversarial samples, this paper proposes an improved method for selecting feature points within the JSMA framework.

The proposed method begins by sorting all feature points in the saliency map based on their saliency values. It then selects the top 8% (which is an empirical value) of feature points, which represent the highest saliency values as the target set to add a one-step disturbance θ. The selection of feature points is shown in Formula (14).

(14) $$(p_{1},\cdots ,p_{n})=[sort(S(x,t)[i])]\times \mathrm{Top}8\%$$

Add the disturbed feature points to the original sample x to obtain the adversarial sample $x^{adv}$. In addition, considering that JSMA is limited by $L_{0}$, it will be easy to be recognized by human eyes if the disturbance added to the feature points is too large. Therefore, in order to enhance the concealment, this paper introduces $L_{\infty }$ to limit ε, that is, the maximum disturbance added by each significant point does not exceed ε, as shown in Formula (15). Then, the adversarial sample is generated iteratively through Formula (16).

(15) $$||x_{i}^{adv}-x_{i}|{|}_{\infty }\leq \varepsilon$$

(16) $$x_{n}^{adv}=Clip_{\varepsilon }[{\left(x_{i,n-1}^{adv}\right)}_{i\in \{p_{1},\ldots p_{n}\}}+\theta ]$$

where $x_{n}^{adv}$ is the countermeasure sample generated through n iterations, and Clip(·) is the clipping function to limit the disturbance size. The overall process of the BPNT-JSMA algorithm is shown in Algorithm 1.
**Algorithm 1** BPNT-JSMA adversarial sample attacks**Input:** Normal signal sample x, DNN model F, Other types of c, Single step disturbance size θ, Single data point disturbance limit ε, Total disturbance limit λ, Iteration number n.**Output:** Adversarial samples $x^{adv}$.1:   Input the sample x into the model F, and return the score r of each category $F_{r}(x)(r\in [1,n])$ 2:   Calculate the Jacobian matrix $J(F_{r}(x))$ of each category according to $F_{r}(x)$ and Formula (9)3:   Calculate the characteristic saliency diagram S(x,c) of other categories of c according to Formula (12) and $J(F_{r}(x))$ 4:   While saliency graph S(x,c) is not empty5:     For (i=0,i<m,i++) 6:       Select the significant feature point $\left\{p_{1},\ldots p_{n}\right\}$ from S(x,c) according to Formula (13) to add the disturbance θ 7:       Generate adversarial samples according to Equation (15)8:       If $F(x^{adv})=c$ 9:         Return adversarial sample $x^{adv}$ 10:     Else11:       If single point disturbance θ<ε and total disturbance <λ 12:        Continue13:     Else14:        Failure to generate adversarial samples15:        Break for16:   End for17:  End while

## 4. Experimental Results and Analysis

### 4.1. Experimental Setup

#### 4.1.1. Data Set

In this study, four frequency-hopping signals are generated as experimental data samples through Matlab R2020a software simulation. In order to better verify the classification recognition accuracy under multiple frequency-hopping signals, the modulation mode of the frequency-hopping signal samples is BPSK, the number of frequency-hopping points is 64, the bandwidth of the frequency-hopping band is 1.6 MHz, the signal sampling rate is fs = 12.8 MHz, the Gaussian white noise is added at the same time to simulate the actual channel conditions, and the range of the signal-to-noise ratio is from −20 dB to 18 dB, with a step of 2 dB. The data set generates 1600 samples at each signal-to-noise ratio, that is, 400 samples for each frequency-hopping signal, and a total of 32,000 samples are generated. In order to ensure the effectiveness of model training and validation, the data set is processed by a random partition strategy: 70% as the training set, 20% as the validation set, and the remaining 10% as the test set. The parameters of frequency-hopping signals in the data set are shown in Table 1.

#### 4.1.2. DNN Model

The performance of the target model in adversarial attacks is crucial in reflecting the effectiveness of the attack. If the target model itself has low accuracy in recognition, then the attack loses its significance. Given that VTCNN2 is a convolutional neural network specifically designed for processing time-series data, it can better capture temporal variations compared to a traditional CNN, making it more suitable for handling frequency-hopping signals. The CLDNN, as the only network model that combines CNNs and Long Short-Term Memory (LSTM) networks, is capable of simultaneously considering both temporal and spatial information in signals. It retains information from previous inputs when processing sequential data, thereby maintaining long-range dependencies and avoiding issues such as vanishing or exploding gradients, which are commonly encountered by traditional network architectures such as DNNs and CNNs when dealing with long sequences. This gives CLDNN a significant advantage in the recognition and adversarial generation of frequency-hopping signals. Meanwhile, ResNet employs residual connections, enabling more efficient training of deeper networks and addressing the vanishing gradient problem that can arise during the training of deep networks. Considering the differences in features and parameters between frequency-hopping signals and images, this paper adopts three classification models: VTCNN2 [22], CLDNN [23], and ResNet [24]. Each model is trained for 500 epochs with an initial learning rate of 0.001, and the learning rate is halved if the validation loss does not decrease for five consecutive evaluations.

To ensure that the data set of multi-frequency-hopping signals is compatible with the DNN model, this paper optimizes and adjusts the network structure of the VTCNN2 model for the specified data set. The model consists of one input layer, four convolutional layers, four pooling layers, four dropout layers, two fully connected layers, and one output layer. By incorporating multiple convolutional layers to extract features at different levels, the model is better equipped to understand and recognize complex data content. The addition of pooling layers reduces computational complexity and accelerates the training speed of the model. Furthermore, the inclusion of dropout layers effectively prevents overfitting and enhances the model’s generalization ability. The specific architecture of the VTCNN2 model is presented in Table 2.

#### 4.1.3. Evaluation Index

To effectively evaluate the algorithm proposed in this paper, the evaluation method outlined in reference [25] is enhanced and examined from three dimensions: the concealment of adversarial samples, the effectiveness of the attack, and the efficiency of the attack.

1. Attack effectiveness

This paper divides attack effectiveness into three dimensions: attack success rate (ASR), Average Confidence of Adversarial Class (ACAC) and Average Confidence of True Class (ACTC).

From the perspective of countermeasure samples, the ASR is defined as the ratio of the number of countermeasure samples that successfully induce the target model to make incorrect predictions to the total number of samples. This metric directly reflects the effectiveness of the attack method, with a value range between 0 and 1. A value closer to 1 indicates a higher attack success rate. If the total number of test samples is M and the number of samples of the successful attack model is N, the formula for calculating the ASR is

(17) $$\mathrm{ASR}=\frac{N}{M}\times 100\%$$

From the perspective of the decision-making mechanism, the average confidence of the adversarial category refers to the mean confidence level of the target model across all potential categories, excluding the true category, in relation to a batch of adversarial samples that have successfully executed an attack. This value ranges from 0 to 1; a higher value indicates greater confidence of the model in classifying the sample into incorrect categories, thereby reflecting a more effective attack. Let the confidence of the attack category for a single sample be denoted as a, and let the total number of samples be M. Then, the average confidence of the adversarial category, denoted as ACAC, is defined as

(18) $$\mathrm{ACAC}=(\frac{1}{M}\sum_{1}^{M}a)\times 100\%$$

Similarly, the average confidence of the real category refers to the mean confidence of the model regarding the true category of a batch of successfully attacked adversarial samples, with a value range between 0 and 1. A higher value indicates that the model has greater confidence in correctly identifying the sample as belonging to the real category, suggesting a weaker attack effect of the adversarial samples. Let b represent the confidence of the model in the correct category for a single sample, and let M denote the total number of samples. Then, the ACTC can be expressed as follows:

(19) $$\mathrm{ACTC}=(\frac{1}{M}\sum_{1}^{M}b)\times 100\%$$

2. Attack efficiency

When evaluating attack efficiency, the ATC for generating each adversarial sample serves as the key indicator. This metric directly reflects the efficiency of the attack; a smaller ATC value indicates that more adversarial samples can be generated within the same time-frame. If the number of successful attack samples is denoted as N and the total time taken is T, then the ATC can be calculated as follows:

(20) $$\mathrm{ATC}=\frac{T}{N}$$

3. Adversarial sample concealment

The concealment of adversarial samples is quantified by the difference between adversarial samples and original samples and is measured using structural similarity (SSIM). The main parameters of SSIM include the brightness $l(x,x^{adv})$, contrast $c(x,x^{adv})$, and structure $s(x,x^{adv})$ of two kinds of pictures. The frequency-hopping signal data used in the experiment can be regarded as a single channel image, and the brightness l and contrast c results are both 1, so only the structural similarity index SSIM needs to be calculated. The calculation formula is as follows:

(21) $$\mathrm{SSIM}(x,x^{adv})={\left[l(x,x^{adv})\right]}^{\alpha }\cdot {\left[c(x,x^{adv})\right]}^{\beta }\cdot {\left[s(x,x^{adv})\right]}^{\gamma }$$

(22) $$s(x,x^{adv})=\frac{\sigma_{x_{1}x_{2}}+C}{\sigma_{x_{1}}\sigma_{x_{2}}+C}$$

where $\sigma_{x_{1}}$ and $\sigma_{x_{2}}$ are the standard deviations of $x_{1}$ and $x_{2}$, respectively, $\sigma_{x_{1}x_{2}}$ is the covariance of $x_{1}$ and $x_{2}$, and C is a constant to avoid the denominator approaching zero. The SSIM ranges from 0 to 1, with values closer to 1 indicating greater similarity between the adversarial sample and the original sample.

### 4.2. Experimental Results and Analysis

#### 4.2.1. Target Model Training Settings

**Figure 2:**
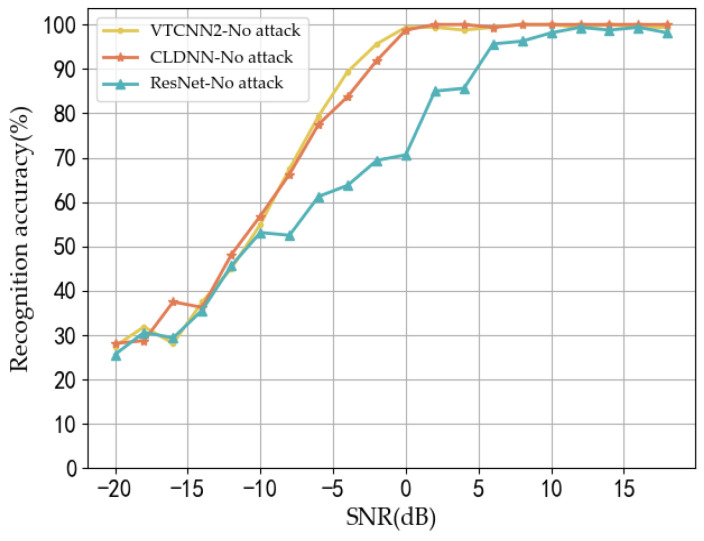
Recognition accuracy of the three models on clean data.

Considering the characteristics of the signal samples, as well as the model parameters and recognition performance under normal conditions, we selected three DNN models—VTCNN2, CLDNN, and ResNet—as the neural network models for evaluating the performance of the comparison algorithms. In the experimental results presented in Figure 2, we assessed the classification recognition accuracy of these three models under non-attack conditions. It was observed that as the SNR gradually increased, the recognition accuracy of all three models exhibited a positive upward trend and eventually stabilized. Among them, the VTCNN2 and CLDNN models demonstrated superior recognition performance, significantly outperforming the ResNet model. In scenarios with a low signal-to-noise ratio, the noise power is considerably greater than the power of the signal itself, leading to severe distortion of the signal waveform. This distortion adversely affects the model’s recognition capability, resulting in generally low accuracy in recognition.

When the recognition rate of the model for the signal under low-SNR conditions is insufficient, it becomes challenging to effectively evaluate the attack’s impact and verify the effectiveness of the attack method. In contrast, when the SNR exceeds 2 dB, the recognition accuracy of both the VTCNN2 and CLDNN models utilized in this experiment remains stable above 95%. Additionally, the ResNet model achieves a recognition accuracy of approximately 80%, which adequately meets the target model’s requirements for resilience against attacks. The recognition accuracy of the three models under normal conditions is shown in Figure 2.

#### 4.2.2. Analysis of Experimental Results

In order to better compare and analyze the methods in this paper, FGSM [9], I-FGSM [10], MI-FGSM [11], PGD [14] and NT-JSMA [8] methods are selected, and these methods are set as non-target-attack mode. The experimental results of the three models are shown in Table 3, Table 4 and Table 5.

##### Attack Success Rate (ASR)

**Figure 3:**
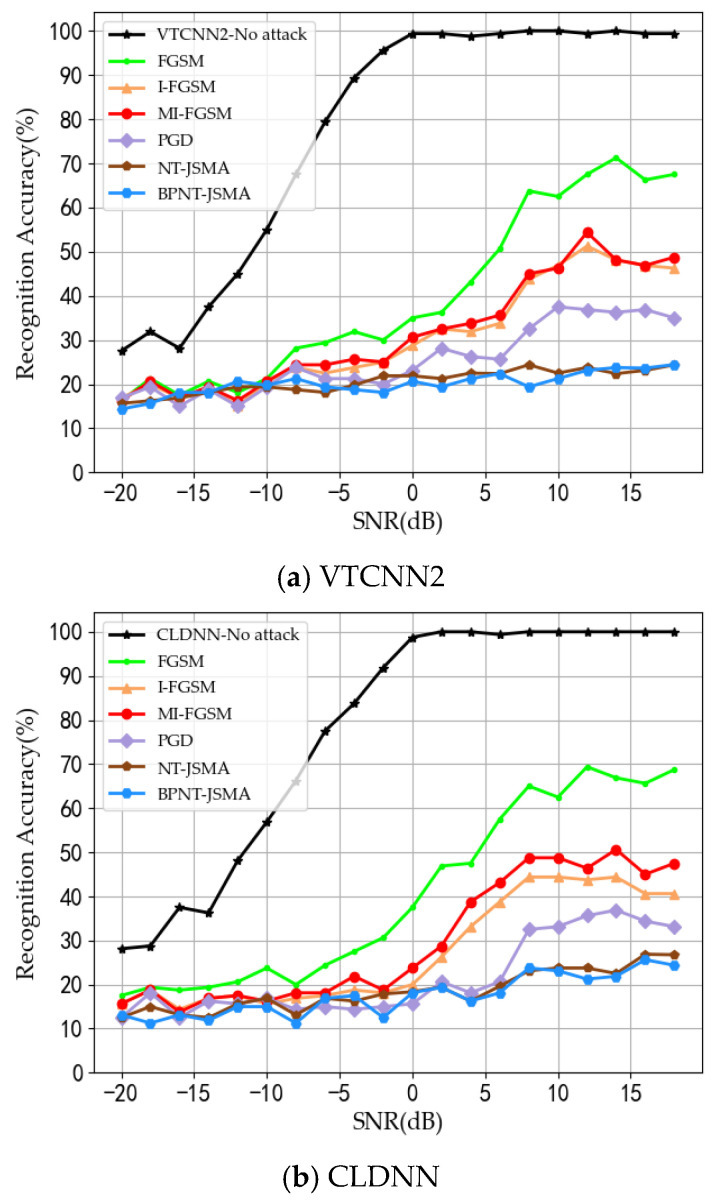

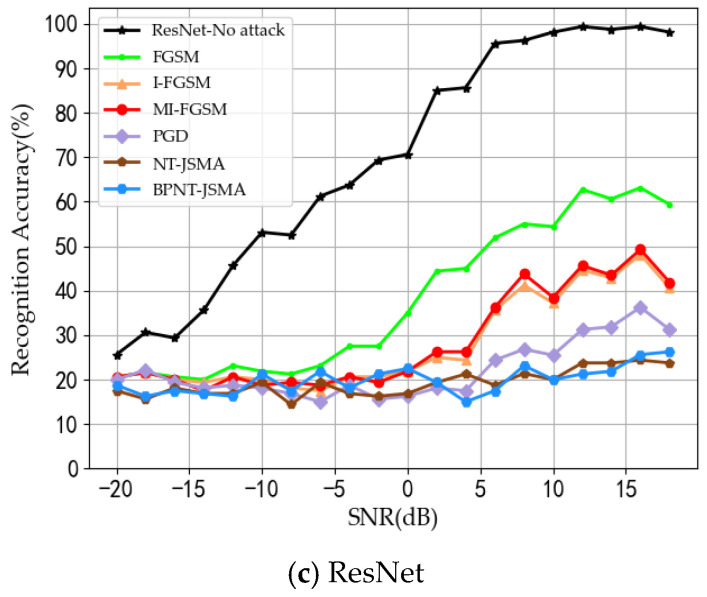
Recognition accuracy of the three models.

The attack success rate can be quantified by the recognition accuracy of the models at different signal-to-noise ratios; a higher attack success rate corresponds to a lower accuracy of the model. The recognition accuracy graphs of the three models at different signal-to-noise ratios are shown in Figure 3.

As shown in Table 3, Table 4 and Table 5, regardless of the attack method employed, the success rate of the three models exceeds 59%. Among these methods, JSMA demonstrates the highest attack efficacy, with a success rate surpassing 83%. Based on Figure 3, it is evident that from −20 dB to 18 dB, as the SNR increases, the model’s recognition accuracy tends to improve, stabilizing around 8 dB. The comparison of the algorithms reveals the following: (1) The effectiveness of the same attack varies across different models, with FGSM performing the worst. A possible reason is that FGSM, as a single-step attack, may produce incorrect gradient directions for generating adversarial perturbations in highly nonlinear deep models. In Figure 3a, at 8 dB, FGSM only reduces the model’s recognition rate by 35%. (2) I-FGSM performs better than FGSM, reducing the VTCNN2 model’s recognition accuracy by 50% at 10 dB. Although MI-FGSM theoretically improves the attack by introducing a momentum term to refine the gradients, its effectiveness is nearly identical to that of I-FGSM, as seen in the figure. In some cases, such as with the CLDNN model, MI-FGSM even underperforms I-FGSM, possibly due to structural differences between models affecting the output. (3) The PGD algorithm combines gradient information with projection operations, primarily utilizing gradient information to iteratively adjust the samples while ensuring that the adversarial samples remain within a certain allowable range after each step. This effectively increases the intensity of the attack. As shown in Figure 3a,b, the model’s recognition rate under PGD attack decreases to below 40%. (4) The algorithm proposed in this paper generates adversarial samples by adding perturbations to key feature points based on the salient feature maps of the samples. To enhance efficiency, a method of batch selecting the most salient points is employed, addressing the issue of weak attack capabilities on certain signals due to the limited information content of the signal samples. This approach reduces ineffective perturbations, resulting in more refined perturbations and improved attack effectiveness. At 10 dB, the recognition rate of the VTCNN2 model can be reduced by approximately 75%, which represents an improvement of 25% over FGSM and 15% over MI-FGSM.

##### Average Confidence of True Class (ACTC) and Average Confidence of Adversarial Class (ACAC)

**Figure 4:**
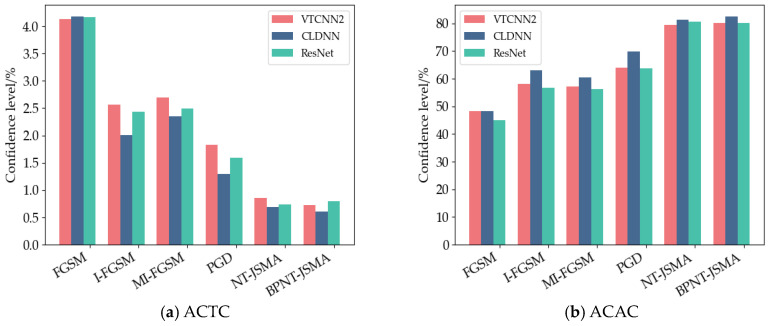
Comparison of ACTC and ACAC in three models.

Figure 4 is a comparison diagram of ACTC and ACAC drawn according to Table 3, Table 4 and Table 5. It can be seen from Figure 4a that the average confidence of any attack method in the correct category is less than 5%, and the values of NT-JSMA and BPNT-JSMA are less than 1%, which shows that in terms of attack effect, the attack based on a saliency graph is stronger than the attack based on gradient. It can be seen from Figure 4b that the average confidence of the confrontation category is above 45%, while the values of the NT-JSMA and BPNT-JSMA attack methods can reach 79%, indicating that the BPNT-JSMA method proposed in this paper will not reduce the attack ability of the original JSMA method. Compared with the other four attack methods, this method still has strong inducement and makes the model decision wrong.

##### Average Time Consumption (ATC)

**Figure 5:**
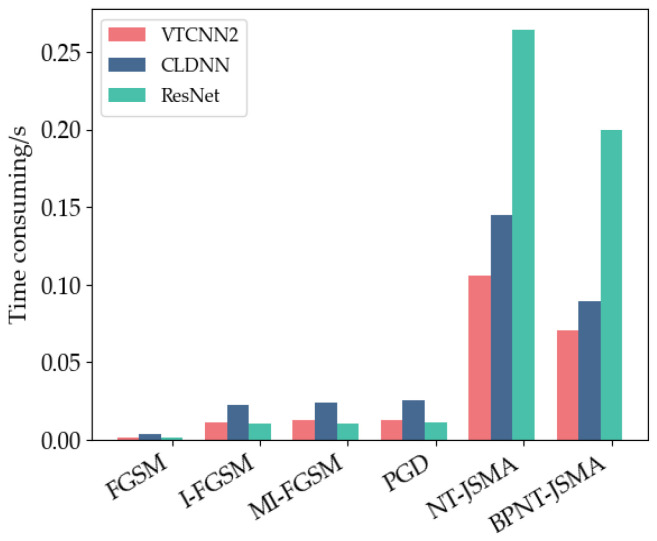
Generation time of single countermeasure sample.

The consumption time required for generating a single countermeasure sample is illustrated in Figure 5. As shown in the figure, despite the earlier analysis indicating that the FGSM attack exhibits the poorest performance, it generates samples the fastest due to its nature as a non-iterative, one-step attack. In contrast, FGSM, I-FGSM, MI-FGSM, and PGD are all iterative attacks with the same number of iterations, resulting in longer running times; however, the average time required to generate a single sample among these methods is not significantly different. The JSMA method requires the calculation of the Jacobian matrix for each pixel of the input sample and necessitates multiple selections of the most resistant pixel for perturbation, making it the most time-consuming attack method. Although the BPNT-JSMA method proposed in this paper has a longer ATC compared to FGSM, I-FGSM, MI-FGSM, and PGD, it is significantly faster than the original JSMA method for selecting feature points to attack, as it selects feature points in batches after calculating the saliency map.

##### Structural Similarity (SSIM)

**Figure 6:**
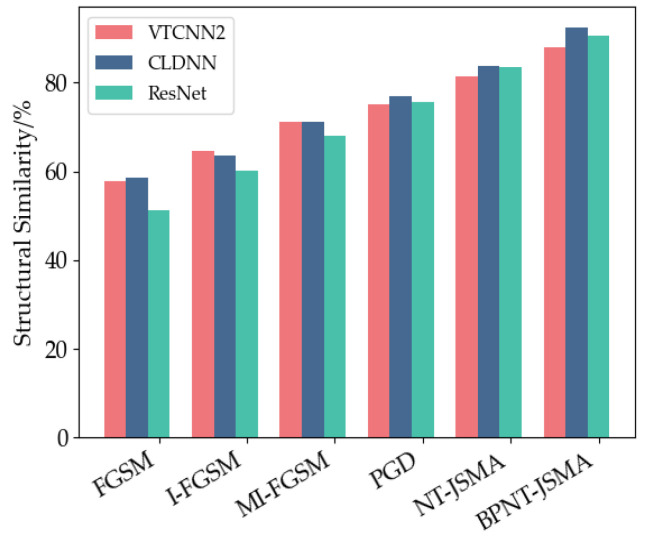
Structural similarity of countermeasure samples.

When adversarial samples exhibit a high similarity to the original data in the feature space, the model is often more easily misled. Additionally, the similarity between samples reflects the model’s vulnerability in specific regions; a high similarity indicates that even minor perturbations can lead to significant output changes, thereby increasing the success rate of the attack. It can be seen from Table 3, Table 4 and Table 5 that the structural similarities between the adversarial samples generated by the FGSM, I-FGSM, MI-FGSM, and PGD attack methods and the original samples are approximately 50%, 60%, 70%, and 75%, respectively. In contrast, the structural similarity between the adversarial samples generated by the NT-JSMA method and the original samples is about 83%; it is positively correlated with the variation in attack success rate. This discrepancy can be attributed to the fact that gradient-based attacks focus more on global gradient information, applying perturbations in the direction of the gradient. In contrast, the JSMA emphasizes local pixel information, allowing for precise localization of key pixels. Consequently, the attack target can be achieved with smaller perturbations, resulting in adversarial samples that exhibit higher similarity to the original samples. Notably, the proposed BPNT-JSMA method achieves a maximum structural similarity of 92% when generating adversarial samples, which is significantly better than that of other methods. This demonstrates that the BPNT-JSMA method can produce adversarial samples that are both highly similar to and less detectable than the original samples while effectively maintaining the attack’s efficacy. See Figure 6.

## 5. Conclusions

To address the security issue where deep learning models for frequency-hopping signal classification and recognition are vulnerable to non-targeted attacks, this paper proposes a novel BPNT-JSMA adversarial sample generation method. In contrast to the traditional NT-JSMA anti-attack method, the proposed approach batch-selects the key feature points that most significantly affect the model’s recognition results, based on the saliency map of frequency-hopping signals, to introduce perturbations. Additionally, it incorporates a perturbation limit, which is absent in the NT-JSMA method, to constrain the magnitude of the perturbations. Experimental results demonstrate that the proposed method significantly improves the speed of adversarial sample generation while enhancing the concealment of the adversarial samples, all without compromising the success rate of the non-targeted JSMA on DNN models. Future research can explore the following directions: (1) The experiments in this paper were conducted in a white-box environment without integrating black-box attacks. In real-world battlefield scenarios, information related to the target model is often unknown to communicators. Therefore, future research could focus on attack methods in black-box environments. (2) While the proposed method shows significant improvements in generation efficiency compared to the original JSMA method, there is still a performance gap compared to gradient-based attack methods. Further efforts to enhance the generation efficiency of adversarial samples while maintaining attack success rates could be a promising avenue for future research.

## Figures and Tables

**Table 1:** Simulation parameters of frequency-hopping signals.

Signal Type	Hopping Speed	Carrier Frequency	Label
Signal 1	500	0–1.6	0
Signal 2	500	1.6–3.2	1
Signal 3	1000	0–1.6	2
Signal 4	1000	1.6–3.2	3

**Table 2:** VTCNN2 model.

Layers	Output Shape
Conv2D	(512,512)
Maxpooling2D	(256,512)
Dropout	(256,512)
Conv2D	(256,512)
Maxpooling2D	(128,512)
Dropout	(128,512)
Conv2D	(128,128)
Maxpooling2D	(64,128)
Dropout	(64,128)
Conv2D	(64,128)
Maxpooling2D	(32,128)
Dropout	(32,128)
Flatten	4096
Dense	256
Dense	4

**Table 3:** VTCNN2 model results.

	ASR/%	ACTC/%	ACAC/%	ATC/s	SSIM/%
FGSM	60.09	4.13	48.30	0.0017	57.87
I-FGSM	69.31	2.56	58.26	0.0116	64.49
MI-FGSM	68.44	2.70	57.27	0.0125	70.10
PGD	74.56	1.83	63.98	0.0127	75.03
NT-JSMA	83.33	0.85	79.37	0.1061	81.34
BPNT-JSMA	84.76	0.72	80.14	0.0706	88.12

**Table 4:** CLDNN model results.

	ASR/%	ACTC/%	ACAC/%	ATC/s	SSIM/%
FGSM	59.53	4.18	48.31	0.0034	58.63
I-FGSM	72.69	2.01	63.18	0.0227	63.62
MI-FGSM	70.15	2.35	60.59	0.0244	70.28
PGD	78.44	1.30	69.84	0.0252	76.96
NT-JSMA	84.59	0.69	81.40	0.1451	83.85
BPNT-JSMA	85.49	0.61	82.53	0.0893	92.46

**Table 5:** ResNet model results.

	ASR/%	ACTC/%	ACAC/%	ATC/s	SSIM/%
FGSM	62.07	4.17	45.01	0.0015	51.30
I-FGSM	71.97	2.43	56.68	0.0103	60.12
MI-FGSM	71.49	2.49	56.32	0.0103	67.06
PGD	77.90	1.59	63.79	0.0110	75.72
NT-JSMA	85.69	0.74	80.77	0.2644	83.66
BPNT-JSMA	86.03	0.79	80.09	0.1995	90.72

## Data Availability

The data presented in this study are not available due to privacy.
